# Rapid amelioration of anorexia nervosa in a male adolescent during metreleptin treatment including recovery from hypogonadotropic hypogonadism

**DOI:** 10.1007/s00787-021-01778-7

**Published:** 2021-05-09

**Authors:** Jochen Antel, Susanne Tan, Marvin Grabler, Christine Ludwig, Dominik Lohkemper, Tim Brandenburg, Nikolaus Barth, Anke Hinney, Lars Libuda, Miriam Remy, Gabriella Milos, Johannes Hebebrand

**Affiliations:** 1 Department of Child and Adolescent Psychiatry, University Hospital Essen, University of Duisburg-Essen, Essen, Germany; 2 Department of Endocrinology, Diabetes and Metabolism, University Hospital Essen, University of Duisburg-Essen, Essen, Germany; 3 Eating Disorders Unit, Department of Consultation-Liaison Psychiatry and Psychosomatic Medicine, University Hospital of Zurich, University of Zurich, Zurich, Switzerland; 4 Research Unit, Department of Child and Adolescent Psychiatry, Psychosomatics, and Psychotherapy, University Hospital Essen (LVR-Klinikum), University of Duisburg-Essen, Virchowstr. 174, 45147 Essen, Germany; 5 KJP-Forschungsabteilung, Holsterhauser Platz 2, Cranachhöfe, 2. OG, Raum 32, 45147 Essen, Germany

**Keywords:** Anorexia nervosa, Metreleptin, Antidepressive, Hypogonadotropic hypogonadism, Hyperactivity

## Abstract

With this case report we support our medical hypothesis that metreleptin treatment ameliorates starvation related emotional, cognitive and behavioral symptomatology of anorexia nervosa (AN) and show for the first time strong effects in a male patient with AN. A 15.9 year old adolescent with severe AN of eight-month duration was treated off-label with metreleptin. Hyperactivity was assessed with accelerometry. Visual analogue scales (VAS), validated self- and clinician rating scales and lab results tracked changes from baseline to end of the 24-day dosing period and a five-month follow-up. Substantial improvements of mood and eating disorder related cognitions and hyperactivity set in after two days of treatment. During dosing, sub-physiological testosterone and TT3 levels normalized; clinically libido reemerged. Weight did not increase substantially during the dosing period. During follow-up target weight was attained; mood did not deteriorate; hyperactivity ceased. The results substantiate the strong effects seen in female cases and underscore the need for a double-blind placebo-controlled trial to confirm the observed strong, multiple and rapid onset beneficial effects of metreleptin in AN.

**Supplementary Information:**

The online version contains supplementary material available at 10.1007/s00787-021-01778-7.

## Introduction

The rationale for metreleptin treatment of patients with AN includes (1) observation of hypoleptinemia as a biological marker of AN [1–4], (2) hypoleptinemia dependent endocrine adaptations to starvation [5], of which particularly the effects on the hypothalamus–pituitary–gonadal axis have been investigated in both female and male patients with AN [6–9], (3) pronounced effects of realimentation on psychopathology in patients with AN, (4) evidence that the gain in fat mass induced increase in leptin secretion underlies several of the mental and behavioral changes [10], and (5) animal models indicative of strong effects of leptin on starvation related running wheel activity and behaviors assessed in depression models [10, 11]. Leptin reaches all regions of the brain, so that a wide range of central effects appears possible [12, 13].

In our recent case series [14] three female patients with acute AN were treated with metreleptin for six to 14 days. Two of the patients showed a pronounced and beneficial clinical response; the third patient with the lowest body mass index (12.5 kg/m^2^; BMI) responded, too, albeit to a reduced extent. The main findings included: (a) antidepressant effect within two days of metreleptin treatment readily apparent to patients, relatives and therapeutic team, (b) reduced drive for activity, (c) reduced inner restlessness, (d) reduced fear of weight gain, (e) reduced preoccupation with food, (f) self-reported improvements of concentration, and (g) increments in social interaction. Particularly, the antidepressant effect was pronounced and may underlie or contribute to some of the other improvements. Whereas an effect on mental symptoms occurring in the state of starvation had a priori been hypothesized [10], the patients’ decreased perception of fear of gaining weight was unexpected as were their feelings of being less subjugated by their eating disorder. Hypothetically, metreleptin treatment may open the cage [15] by alleviating an addictive-like state related to the metabolic adaptation to starvation and its effects on the central nervous system in thus predisposed patients [10].

## Case report

Fifteen year old F initially presented with a BMI of 14.1 kg/m^2^ (< 1st BMI sex- and age-matched percentile) after a rapid weight loss of 12 kg within 2.5 months (premorbid BMI 18.1 kg/m^2^) fulfilling DSM-5 diagnostic criteria for the restricting type of AN; F skipped meals and replaced regular ‘unhealthy’ food with fruits and vegetables. Mood and liveliness had deteriorated over the last six months. F had been active in team sports but had increasingly resorted to daily endurance training. F’s weight as a child had been above average (annual documentation of measured height and weight between ages 0 to 5; BMI percentiles between 61 and 81; photos during later childhood suggest overweight). His protruding belly had been of concern to him from the age of seven years on; upon referral he perceived himself as chubby. During his first inpatient treatment of two months duration F gained nine kg to be discharged at 52.2 kg (17.0 kg/m^2^). Due to instable mood, persistent inner tension, restrictive eating and renewed weight loss (50.5 kg) he was readmitted after only two weeks. After seven weeks F discharged himself (57 kg); a pronounced body image disturbance persisted.

After only ten days he sought renewed admission (53.7 kg) due to suicidal ideation and excessive hyperactivity. The clinical situation nevertheless deteriorated substantially and was characterized by stagnation of weight gain (oscillating around 52 kg; Suppl. Table 1), severe hyperactivity, depression and AN specific cognitions and manipulative behavior. Repeated episodes of hyperactivity lasted 2–4 days in form of brisk walking during the entire day (up to 70,000 steps/d; see “Methods” section) within a secured inner courtyard in addition to compulsive exercise conducted in his room and bathroom (self-reported 2000 jumping jacks and 200 pushups daily). Such episodes were followed by two days of total exhaustion and severe depression, during which he remained in bed. On two occasions he threatened to commit suicide by jumping from a bridge entailing large scaled police searches; upon the second time he also conveyed extortion demands to be met by parents and treatment team to circumvent weight gain and therapeutic restrictions. In light of the severe clinical symptomatology both patient and parents agreed to an off-label treatment with metreleptin and provided written informed consent in accordance with the latest version of the Declaration of Helsinki [16].

## Methods

Metreleptin dosages of 3–9 mg/d (3 mg: d1–d2, d4, d8, d13–d20, d22, d24; 6 mg: d3, d5–d7, d11–d12; 9 mg: d9–d10) were applied subcutaneously once daily at 9:30 am. The dosage was tapered prior to discontinuation (no dosing on d21 and d23). Concurrent medication to reduce hyperactivity included diazepam up to 2.25 mg between baseline and d4 and again from d9 to d17. Olanzapine (3 mg/d) was discontinued after d3. Nasogastric feeding was pursued throughout the dosing period with F sometimes additionally eating food orally (Supplementary Table 1).

We used visual analog scales (VAS; [14] to obtain twice daily (morning and evening; presented as means) self-ratings for eating disorder related cognitions, emotions and behaviors. Clinician- and self-ratings of depression were obtained with Childhood Depression Rating Scale-Revised [17, 18] and Beck Depression Inventory-II [19, 20]. Self-rated eating disorder related cognitions and behaviors were assessed with Eating Disorder Inventory-2 [21]. Assessments were commenced nine days prior to treatment and continued until post-dosage day 47. Accelerometry (ActiGraph^®^; ActiGraph GT3X (Pensacola, Florida, USA); software version 6.13.3, firmware v1.9.2) was used to count steps. Routine and endocrine (free and total triiodothyronine (fT3, TT3), free and total thyroxine (fT4, TT4), thyroid stimulating hormone (TSH), (bioavailable) testosterone, follicle stimulating hormone (FSH), luteinizing hormone (LH), prolactin, insulin, C-peptide, and leptin, for which composite (leptin and metreleptin) diurnal profiles were obtained two times following application of 3 mg (d22) and 6 mg (d11) of metreleptin, respectively) were measured repeatedly. Glucose levels were monitored continuously from −d1 to d8 (Freestyle Libre 2^®^).

Body composition was determined with Dual Energy X-ray Absorptiometry (DXA) and air displacement plethysmography (Bod Pod^®^; see [22]).

## Results

**Fig. 1:**
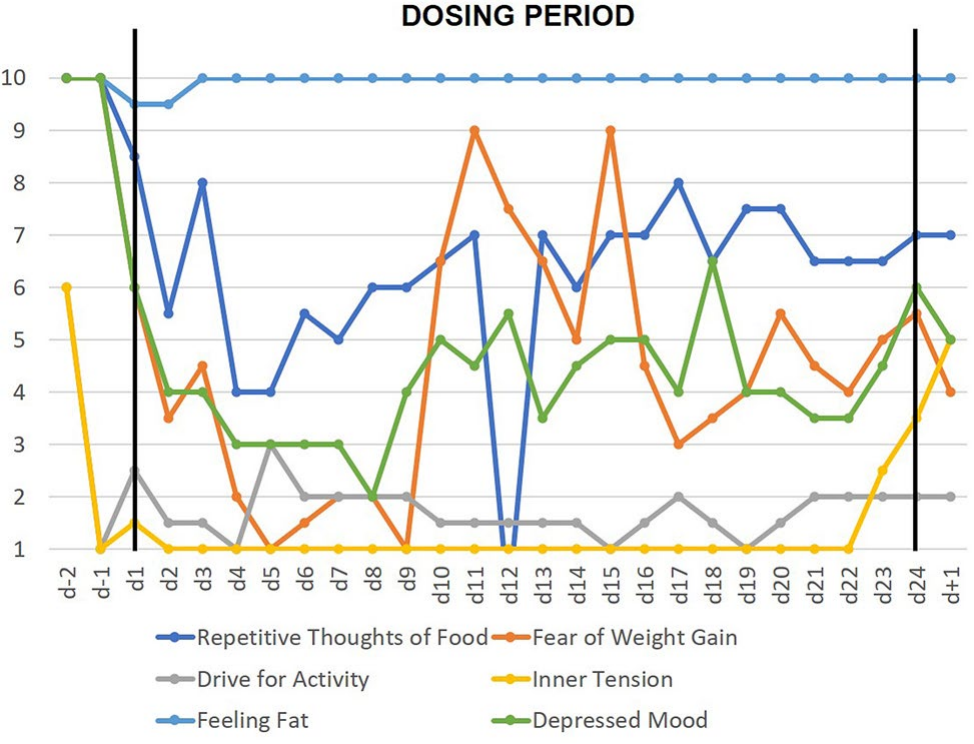
Means of six key cognitions and emotions assessed twice daily with visual analogue scales (range 1–10) prior, during and after the 24-day dosing period

Ratings of several VAS items (‘depressed mood’, ‘repetitive thoughts of food’, ‘fear of weight gain’, ‘inner tension’) dropped within the first days of dosing (Fig. 1). Particularly, mood improved, already rated as better in the evening of the first day of dosing. At 9 mg/d (d9,d10), F’s mood appeared overly buoyant, so that dosing was subsequently maintained at 3 mg/d. Whereas ‘fear of weight gain’ was intermittently ranked as absent (d5 and d9), ‘feeling fat’ was continuously ranked as maximal. F for the first time reported the strong negative effects weight teasing had had on his self-perception during childhood. Throughout dosing his perception of his “fat belly” appeared delusional.

‘Drive for activity’ was continuously ranked as low; dosing had commenced after F had remained in bed throughout a weekend, prior to which he had intermittently walked up to 70,000 steps/d (Supplementary Fig. 1). This self-ranking contrasted with continued pacing and exercise, albeit to a reduced extent. Thus, the step count declined and thereafter remained at about 40,000 steps daily; efforts to further decrease hyperactivity by increments in dosing up to 9 mg or re-initiation of medication with diazepam had no additional effect. While F was not able to relent activity prior to dosing, he was able to sit down for up to 30 min upon request during dosing. F explained his high activity level by his fear of becoming lazy entailing further fattening of his belly. During dosing, reduction of creatinine kinase seemingly mirrored the overall reduced hyperactivity (Supplementary Table 5).

Both clinician and self-rated depression decreased substantially (Table 1). The transition from severe to mild and minimal depression (BDI-II) occurred within 14 and 19 days, respectively. The raw score of the EDI-II dropped from 430 (d1) to a low of 239 (d14; Table 1). Reductions in subscale scores were pronounced for Bulimia, Ineffectiveness, Perfectionism, Asceticism, Maturity fears, Interpersonal distrust, and Impulse control (Supplementary Table 2).

**Table 1:** Clinician (Children’s Depression Rating Scale-Revised (CDRS-R)) and self-rated (Beck Depression Inventory-II (BDI-II) total score, Eating Disorder Inventory-2 (EDI-2) total score; data displayed as raw scores/percentile ranks) data of patient F prior to, during and after the 24-day dosing period

Day	d−2	d−1	d1	d4	d5	d8	d14	d15	d19	d + 2	d + 18	d + 31	d + 32	d + 45	d + 47
CDRS-R raw score/percentile rank		80/100			30/77			29/75			31/80		30/77		
BDI-II	58			47		17		12	10	18				9	
EDI-2 raw score			430	399		288	239		240			260		253	268
EDI-2 percentile rank			≥ 95	≥ 95		≥ 95	85		85–90			90–95		90–95	≥ 95

**Fig. 2:**
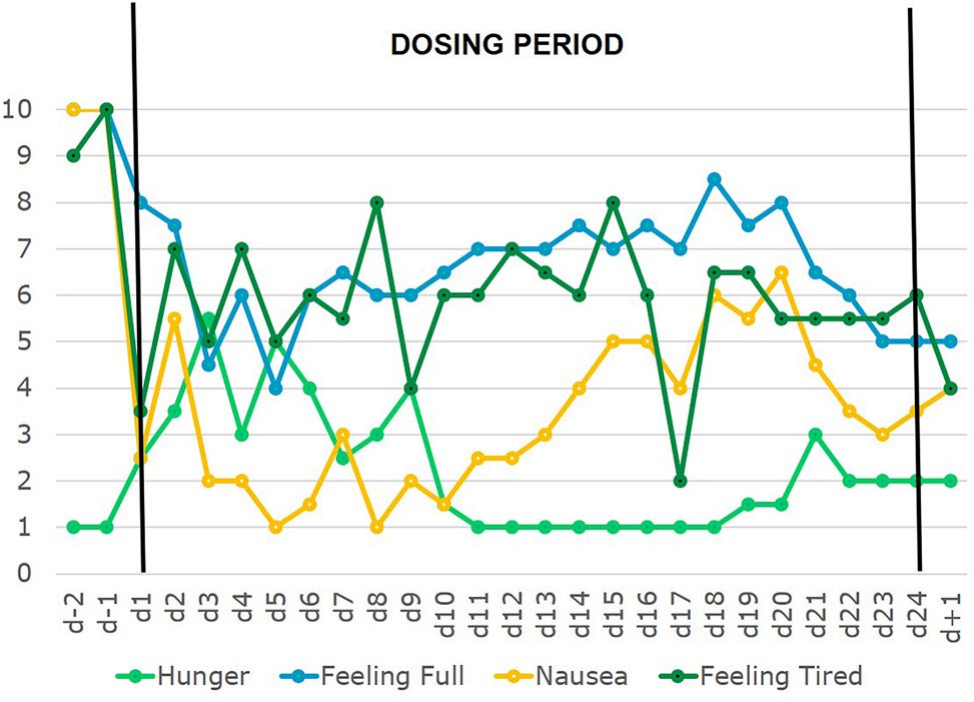
Means of four self-ranked safety/physiological parameters assessed twice daily with visual analogue scales (range 1–10) prior, during and after the 24-day dosing period

The treatment team unsuccessfully exerted extensive efforts including nasogastric feeding to induce weight gain during dosing (Supplementary Table 1); AN related manipulative behavior persisted. VAS-rankings of ‘hunger’ and ‘appetite’ increased over a ten-day period after initiation of dosing, ‘feeling full’ and ‘nausea’ decreased (Fig. 2); the pressure of the treatment team to gain weight and increments in prescribed total daily energy intake per nasogastric feeding confound the assessment of hunger/appetite (Supplementary Table 1).

At d4 F reported being able to concentrate enough to read again; he also stated being able to remember the contents until the next day. Social interactions with therapeutic team and parents improved substantially (Supplementary Table 3). F became much more amenable to psychotherapy; his introspective awareness increased. During the initial dosing period, F was frequently tired and slept longer in the morning. After a week, tiredness was no longer observed, but the self-reported VAS item ‘feeling tired’ oscillated continuously during the dosing period (Fig. 2).

**Fig. 3:**
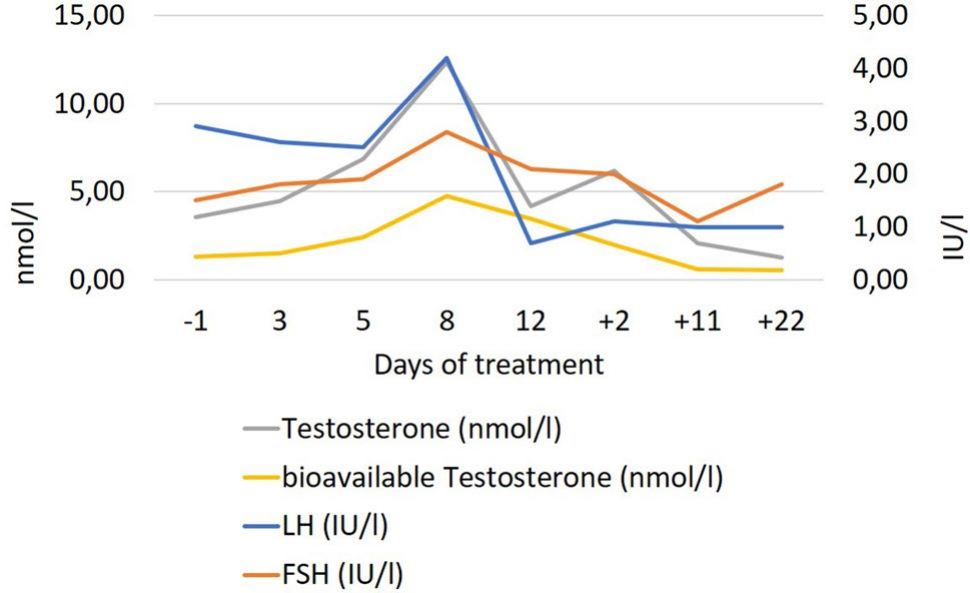
Hormone levels of the pituitary–gonadal axis prior to, during and after the 24-day dosing period (normal ranges: testosterone: 5.0–29.2 nmol/l; LH: 1.0–7.IU/l; FSH: 1.4–7.5 IU/l)

**Fig. 4:**
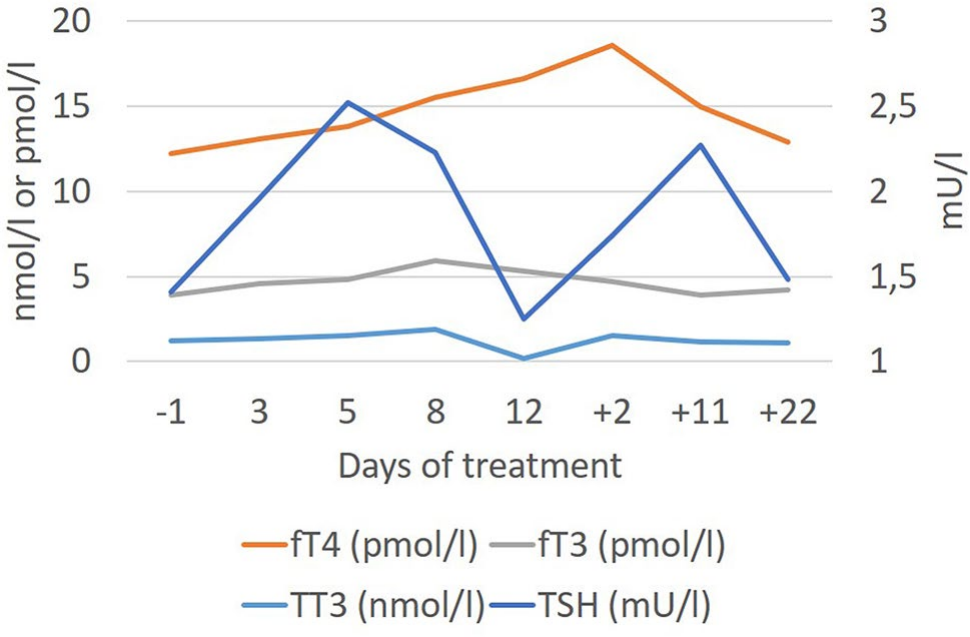
Hormone levels of the pituitary–thyroid axis prior to, during and after the 24-day dosing period (normal ranges: ft3: 4.2–7.47 pmol/l; ft4: 10.57–22.62 pmol/l; TT3:1.31–2.9 pmol/l; TSH: 0.48–4.17 mU/l)

F proudly talked about three female patients who wished him to contact them at d13. F explained that an interest in sex had returned (Supplementary Table 3) and voiced concerns that it would again disappear after end of dosing. In line with the reemergence of libido, he no longer scored in the BDI-II item ‘loss of interest in sex’ (drop from 3 to 0 between d8 and d15). At baseline, the patient presented with hypogonadotropic hypogonadism with marked testosterone deficiency. During treatment, testosterone levels rapidly normalized (Fig. 3). Concomitant to dose reduction to 3 mg/d as of d13 testosterone concentration fell to baseline levels. Changes of the pituitary–thyroid-axis also occurred (Fig. 4). Initially, the patient showed the typical constellation of non-thyroidal illness with low levels of fT3/TT3 and TT4 with inadequately normal TSH. Transient normalization of thyroid hormones occurred during dosing. Prolactin levels dropped after onset of metreleptin treatment (Supplementary Table 5).

Continuous glucose monitoring (nine days) detected two episodes of short, mild hypoglycemia defined as glucose levels below 70 mg/dl at baseline. Neither mild nor severe hypoglycemia were observed during the treatment period. (Supplementary Fig. 2). Prior to treatment levels of C-peptide and insulin were increased, then dropped after onset of metreleptin dosing (Supplementary Table 5).

Prior to dosing serum leptin levels were < 0.1 µg/l. Morning (pre-dosage) levels ranged between 1.0 (d3; 3 mg applied on d2) and 4.0 µg/l (d11; 9 mg applied on d10). Peak concentrations were measured two (45.7 µg/l; d22; morning dose 3 mg) to four hours (57.5 µg/l; d11; morning dose 6 mg) after application. After application of 3 and 6 mg of metreleptin the levels dropped over time to levels of 0.1 and 3.2 µg/l in the subsequent mornings prior to renewed dosing (Supplementary Table 4).

Percent body fat (%BF; Bod Pod^®^) had been at 6.9% at initial referral; during dosing (d12) it was 17.5%. DXA measured at d12, too, revealed a slightly lower total fat mass and 15.6%BF.

*Follow-up*: F successfully began to overcome his eating disorder ten weeks after discharge by continuously pursuing realistic goals. His current weight is 58.2 kg (BMI: 18.6 kg/m^2^) seven months post-dosing. He is receiving outpatient psychotherapy; inpatient treatment was not required again. Depression and severe hyperactivity have not recurred. F returned to full-time schooling prior to recent Covid-19 related school closures in Germany.

## Discussion

This case report provides novel endocrine data and extends our initial report of multiple beneficial effects of metreleptin on AN specific emotions, cognitions and behaviour to the first male patient. Overall, the effects were pronounced and readily evident to patient, parents and treatment staff, including members who had no knowledge of the novel treatment.

It again deserves mentioning that both clinician and self-rated mood (BDI-II score dropped from 58 at –d2 to 10 at d19) improved substantially within days, which contrasts with the slower improvement in mainly only clinician rated depression in randomized controlled trials for antidepressants [23]. Solid evidence for an antidepressant effect of fluoxetine in underweight patients with AN is lacking [24]. Metreleptin may well prove to be the first drug with a strong and rapid onset antidepressant effect in AN related depression. Mood was again judged as intermittently overly buoyant (compare with patient A in Milos et al. [14]). In different animal models, leptin has a substantial antidepressant effect [11].

It is not possible to determine to what extent the reduced inner tension and the substantially improved sleep duration/quality observed in F and our female patients [14] contribute to or are dependent on the observed antidepressant effect. Similarly, it is not possible to disentangle the effects of improved mood from the drop in some eating disorder symptoms (see ‘repetitive thoughts of food’ and ‘fear of weight gain’; Fig. 1). The wide central distribution of leptin receptors suggests multiple potential targets to explain the observed effects, including those on appetite and hunger. Thus, the initial increase in self-rated appetite (Supplementary Fig. 3) and hunger (Fig. 2) mirrors previous findings (patient C; Milos et al. [14]), suggesting that in prolonged starvation both may be down-regulated by chronic hypoleptinemia; patient F additionally reported less nausea and fullness soon after initiation of dosing (Fig. 2). In normal weight subjects [25] and in patients with lipodystrophy [26] metreleptin can induce weight loss via a reduced appetite/hunger.

Whereas F self-rated his ‘drive for activity’ as low prior (bed rest during d-2 and d-1) and throughout the dosing period, hyperactivity remained a challenging symptom (see re-initiation of treatment with diazepam). Nevertheless, activity levels stayed well below those observed a few days prior to dosing (Supplementary Fig. 1); it was also evident that F was much better able to control his hyperactivity upon request.

In contrast to ‘fear of weight gain’, ‘feeling fat’ (Fig. 1) did not decrease during the dosing period. F reported both ego-syntonic obsessions/compulsions related to fear of laziness and a delusional body image disturbance. During and after treatment he was, however, much more able to speak of his difficulties seemingly due to an increased introceptive awareness. F and previously reported patient C [14] belong to the most difficult patients with AN treated in our inpatient unit. Both patients had resisted efforts to induce weight gain for weeks prior to dosing and proved very manipulative with respect to AN related behaviors (the high prescribed oral energy intake (Supplementary Table 1) in F must be interpreted accordingly); both had a history of childhood overweight (F)/obesity (C). Due to the reported increments in “hunger” and “appetite” we assume that the difficulties to gain weight during dosing reflect the severity of the underlying eating disorder and not a side effect of metreleptin. Whereas the initial follow-up period seemed to predict a relapse, F started to set small goals ten weeks post-dosing, thus entailing a rapid improvement. Upon last consultation F was no longer overly concerned with his body shape; his diet was perceived as slightly abnormal by his mother; he had to eat breakfast alone. While it is too early to conclude that F has indeed overcome his eating disorder, the mere twelve months duration of AN despite its intermittent severity warrants notice.

In accordance with known effects of leptin on the hypothalamus–pituitary–gonadal and thyroid axes we observed endocrine alterations compatible with the notion that metreleptin reversed the hypoleptinemia induced starvation related effects on the respective axes. F went through a second puberty within a matter of days; the accompanying increase in sexual interest was readily evident to him and provided a boost to his motivation to avoid renewed weight loss. In female patients with AN, the functional normalization of the reproductive axis is seemingly not as rapid. However, both the relatively high percent body fat and the in comparison to other male [9] and female [8] patients comparably high FSH and LH serum levels at baseline suggest that metreleptin may have merely represented the finishing touch to an already advanced maturation of this axis. A dosage effect also merits consideration, because metreleptin was dosed at only 3 mg/d as of d13, after which the initial surge in testosterone secretion was not upheld. Metreleptin seemingly also induced a transient normalization of the hypothalamus-pituitary–thyroid axis (Fig. 4). The observed reduction in prolactin secretion warrants further study to assess its relationship to metreleptin dosing.

In patients with lipodystrophy, hypoglycaemia represents a treatment emergent adverse event of metreleptin in patients concomitantly treated with antidiabetic medications [27]. We for the first time analysed prolonged effects of metreleptin on blood glucose levels via a nine day long continuous monitoring. The results suggest that metreleptin may reduce the risk of starvation induced hypoglycemia in patients with AN. Throughout the 24 day long dosing period adverse effects were not observed.

Whereas we again cannot exclude expectation effects, the consistency of the clinical results and the endocrine effects underscore our previous assessment that these are induced by metreleptin. The suffering of patients with AN as well as their relatives makes it mandatory to aim for a double-blind placebo-controlled trial to generate evidence for the efficacy of metreleptin for treatment of this eating disorder.

## Supplementary Information

Below is the link to the electronic supplementary material.

Supplementary file1 (DOCX 344 KB)

## Data Availability

All directly sharable data are supplied with the supplementary material.
