# Supplementary material for: Rapid amelioration of anorexia nervosa in a male adolescent during metreleptin treatment including recovery from hypogonadotropic hypogonadism
Source: Eur Child Adolesc Psychiatry. 2021 May 9;31(10):1573–9. doi: 10.1007/s00787-021-01778-7 (PMC8106547; doi:10.1007/s00787-021-01778-7)
Supplement: Supplementary file 1 — Supplementary file1 (DOCX 344 KB) [file 787_2021_1778_MOESM1_ESM.docx]

#

Supplementary Material

# Rapid amelioration of anorexia nervosa in a male adolescent during metreleptin treatment including recovery from hypogonadotropic hypogonadism

Dr. Jochen Antel^1^, PD Dr. Susanne Tan^2^, Dr. Marvin Grabler^1^, Dr. Christine Ludwig^1^, Dominik Lohkemper^1^, Dr. Tim Brandenburg^2^, Dr. Nikolaus Barth^1^, Prof. Dr. Anke Hinney^1^, Prof. Dr. Lars Libuda^1^, Dr. Miriam Remy^1^, Prof. Dr. Gabriella Milos³, Prof. Dr. Johannes Hebebrand^1^

^1^ Department of Child and Adolescent Psychiatry, University Hospital Essen, University of Duisburg-Essen, Essen, Germany

^2^ Department of Endocrinology, Diabetes and Metabolism, University Hospital Essen, University Duisburg-Essen, Germany

³ Eating Disorders Unit, Department of Consultation-Liaison Psychiatry and Psychosomatic Medicine, University Hospital of Zurich, University of Zurich, Zurich, Switzerland

Corresponding author: Dr. rer. nat. Jochen Antel

Research Unit

Department of Child and Adolescent Psychiatry, Psychosomatics, and Psychotherapy

University Hospital Essen (LVR-Klinikum)

University of Duisburg-Essen, Germany

Virchowstr. 174

D-45147 Essen

Office:

KJP-Forschungsabteilung

Holsterhauser Platz 2

Cranachhöfe

2. OG, Raum 32

D-45147 Essen

Tel.: +49 (201)-438755-202

E-Mail: jochen.antel@uni-due.de

**Supplementary Table 1:** Body weight and prescribed energy intake during dosing period (n.d. = not determined).

| **Days** | **Body weight [kg]** | **prescribed kcal per day*** |
| --- | --- | --- |
| d-2 | 50.2 | 4500 |
| d-1 | n.d. | 4500 |
| d1 | **51.8** | 4500 |
| d2 | **51.2** | 4500 |
| d3 | **n.d.** | 4500 |
| d4 | **51.2** | 4500 |
| d5 | **51.2** | 4800 |
| d6 | **51.0** | 4800 |
| d7 | **51.2** | 4800 |
| d8 | **50.9** | 5500 |
| d9 | **51.5** | 5500 |
| d10 | **51.3** | 5500 |
| d11 | **51.8** | 5100 |
| d12 | **52.1** | 5100 |
| d13 | **52.6** | 5100 |
| d14 | **52.8** | 5100 |
| d15 | **52.5** | 5500 |
| d16 | **52.5** | 5500 |
| d17 | **53.0** | 6000 |
| d18 | **53.2** | 6000 |
| d19 | **52.7** | 4500 |
| d20 | **51.5** | 4500 |
| d21 | **52.8** | 4500 |
| d22 | **52.9** | 4500 |
| d23 | **52.5** | 4500 |
| d24 | **52.8** | 4500 |
| d+1 | **52.8** | 4500 |

*Due to pronounced manipulations, the relationship of prescribed to true energy intake is unclear; measured body weights must also be interpreted with caution. The pronounced hyperactivity likely required a high energy intake.

**Supplementary Table 2:** EDI-2 subscale scores during and after dosing period (data displayed as raw scores (RS)/percentile ranks (PR))

| **Days** | d1 | | d4 | | d8 | | d14 | | d19 | | d+31 | | d+45 | | d+47 | |
| --- | --- | --- | --- | --- | --- | --- | --- | --- | --- | --- | --- | --- | --- | --- | --- | --- |
| **EDI-2 Subscale** | **RS** | **PR** | **RS** | **PR** | **RS** | **PR** | **RS** | **PR** | **RS** | **PR** | **RS** | **PR** | **RS** | **PR** | **RS** | **PR** |
| Drive for thinness | 42 | ≥99 | 37 | ≥99 | 27 | 95-99 | 29 | ≥99 | 24 | 95-99 | 25 | 95-99 | 22 | 95-99 | 30 | ≥99 |
| Bulimia | 22 | 95-99 | 14 | 95 | 8 | 35 | 7 | 15 | 7 | 15 | 30 | ≥99 | 29 | ≥99 | 7 | 15 |
| Body dissatisfaction | 54 | ≥95 | 54 | ≥95 | 51 | ≥95 | 47 | ≥95 | 47 | ≥95 | 53 | ≥95 | 46 | ≥95 | 52 | ≥95 |
| Ineffectiveness | 57 | ≥99 | 58 | ≥99 | 35 | 95-99 | 21 | 45 | 27 | 75 | 25 | 65 | 27 | 75 | 28 | 80-85 |
| Perfectionism | 27 | 95-99 | 27 | 95-99 | 17 | 55 | 14 | 35 | 14 | 35 | 12 | 15 | 13 | 20 | 14 | 35 |
| Interpersonal distrust | 21 | 60 | 17 | 30 | 19 | 45 | 17 | 30 | 15 | 10-15 | 12 | 1-5 | 11 | 1 | 15 | 10-15 |
| Interoceptive awareness | 55 | ≥99 | 48 | ≥99 | 35 | 95-99 | 24 | 80-85 | 25 | 90 | 31 | 95-99 | 30 | 95-99 | 26 | 90-95 |
| Maturity fears | 39 | ≥95 | 39 | ≥95 | 24 | 75 | 20 | 45 | 25 | 85 | 20 | 45 | 24 | 75 | 24 | 75 |
| Ascetism | 30 | ≥95 | 33 | ≥95 | 19 | 80 | 18 | 70 | 17 | 65 | 17 | 65 | 16 | 60 | 19 | 80 |
| Impulse control | 42 | ≥95 | 35 | 95 | 24 | 60 | 18 | 15 | 19 | 25 | 14 | 5-10 | 15 | 5-10 | 27 | 80 |
| Social insecurity | 41 | ≥95 | 37 | ≥95 | 29 | 90 | 24 | 60 | 20 | 30 | 21 | 40 | 20 | 30 | 26 | 80 |

**Supplementary Table 3:** Observations/comments of members of treatment staff and parents

| **Day of dosing period** | **Treatment staff** | **Parents** |
| --- | --- | --- |
| **D1** | After having walked briskly for the whole day he was asked to stand still. He jokingly replied: Why? I do not see a traffic radar. |  |
| **D1** |  | First phone call in four weeks during which F did not cry. |
| **D2** | His mood has clearly improved; he establishes eye-contact. |  |
| **D3** |  | I walked with him in the courtyard. I can hardly believe the change - I am very happy. He was in a good mood, talked and joked a lot. He laughed for the first time in more than six weeks. He stated enjoying his appetite, having almost eaten a whole piece of pie. He also stated that his urge to move had almost disappeared. He expressed concern of having to walk with me, because he had already walked enough. He thinks metreleptin is helping him substantially and definitely wants to continue the treatment. |
| **D4** | According to F, the urge to walk briskly has disappeared. Prior to the experienced reduction, walking was experienced both in an ego-syntonic and –dystonic way. He had wanted to increase his walking every day; he had tried to circumvent his tiredness and becoming ‘lazy’.  Reduced compulsivity, spends less time running or showering. | Very good mood and makes jokes. |
|  | He enjoys being able to speak of topics other than his eating disorder; this represents a strong change. He is able to experience an interest in other people. | For the first time in months open for topics not related to his eating disorder. |
|  |  | His former personality is re-appearing. Allows for hugging and nevertheless keeps smiling. |
| **D11** | Reports interest in sex. Lewd remarks addressed at female nurses. |  |
| **D13** | Proudly talked about three female patients who wished him to contact them. |  |
| **D15** |  | Thank you for giving me back my son. |

**Supplementary Table 4:** Serum leptin values.

| **Days** | **Hour at blood sampling** | **Leptin [µg/l]** |
| --- | --- | --- |
| d-2 | 09:00 | < 0.1 |
| d1 | 13:30 | 25.9 |
| d3 | 09:30 | 1.0 |
| d3 | 13:30 | 75.9 |
| d5 | 09:30 | 1.3 |
| d5 | 13:30 | 55.3 |
| d8 | 09:15 | 3.2 |
| d10 | 13:30 | 53.5 |
| d11 | 09:00 | 4.0 |
| d11 | 11:10 | 41.5 |
| d11 | 10:30 | 15.4 |
| d11 | 13:10 | 57.5 |
| d11 | 16:10 | 50.5 |
| d12 | 09:00 | 3.2 |
| d22 | 09:30 | 2.5 |
| d22 | 10:30 | 28.3 |
| d22 | 11:30 | 45.7 |
| d22 | 13:30 | 32.1 |
| d22 | 16:30 | 19.6 |
| d23 | 09:30 | 0.1 |
| d+8 | 10:39 | <0.1 |
| d+11 | 09:00 | 1.9 |

**Supplementary Table 5:** Serum levels of creatine kinase (CK), C-peptide, insulin, prolactin and sex hormone binding globulin (SHBG)

|  | **Days** | | | | | | |  |
| --- | --- | --- | --- | --- | --- | --- | --- | --- |
| **Analytes** | **d-1** | **d3** | **d5** | **d8** | **d12** | **d+2** | **d+11** | **Normal ranges** |
| CK [U/l] | 221 | 170 | 156 | 118 | 146 | 231 | 176 | <180 |
| Prolactin [ng/ml] | 11.3 | 11 | 4.5 | 2.9 | 4.9 | 7.6 | 8.2 | 3.2-13.5 |
| Insulin [uU/ml] | 11.2 | 17.6 | 23.3 | <2.0 | 3.5 | 10.5 | <2.0 | 3.6-29.1 |
| C-Peptide [ng/ml] | 3.54 | 3.8 | 5.65 | 1.75 | 1.09 | 1.89 | 0.538 | 0.9-6.9 |
| SHBG [nmol/l] | 37.7 | 43.2 | 49.6 | 48.6 | 45 | 50.3 | 61.8 | 10-74 |

## Supplementary Figures

Predosing and dosing period up to d16

**Supplementary Fig. 1:** Step counts as measured with accelerometer (ActiGraph®). Data not to be taken at face value due to intermediate non-adherence (time protocols for wearing of accelerometer incomplete); prior to dosing patient experienced waxing and waning of both hyperactivity (e.g. d-9/d-8) and daytime bedrest (d-2/d-1). During dosing daily step counts were similar; days spent in bed did not reoccur. Accelerometry was discontinued at d17.


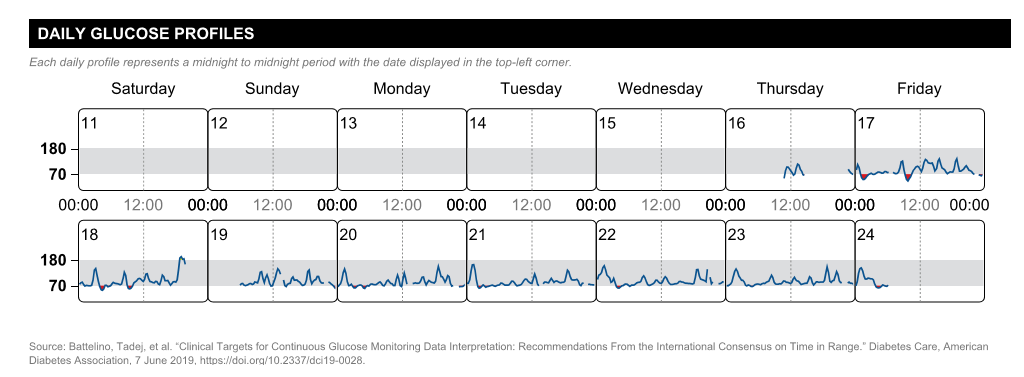


**Supplementary Fig. 2:** Effects of metreleptin on blood glucose during the first week of dosing.

**Supplementary Fig. 3:** Appetite (1 = no appetite) assessed twice daily with visual analogue scales (range 1-10) prior (d-2, d-1), during (d1 to d24) and after the 24-day dosing period.

## Supplementary information: laboratory methods

IDS-iSYS Cortisol, follicle-stimulating hormone (FSH), free triiodothyronine (FT3), free thyroxine (FT4), luteinizing hormone (LH), prolactin, testosterone, total T3 (TT3) and total T4 (TT4) were determined on serum samples with the Siemens ADVIA Centaur® immunoassay-system (Siemens Healthineers, Erlangen, Germany) by chemiluminescence with following detection range, inter-assay variation and intra-assay variation:

Cortisol: 13.8 – 2069 nmol/l, 4.2 % and 6.0 %

TT4: 3.9 – 387 nmol/l, 3.2 % and 4.6 %

TT3: 0.15 – 12.3 nmol/l, 3.2 % and 1.3 %

FT4: 1.3 – 155 pmol/l), 3.3 % and 4.0 %

FT3: 0.3 – 30.8 pmol/l), 3.1 % and 4.1 %

LH: 0.07 – 200 mIU/ml, 3.0 % and 2.9 %

FSH: 0.3 – 200 mIU/ml, 2.9 % and 2.7 %

Testosterone: 0.35 – 52.1 nmol/l), 6.2 % and 4.7 %;

Prolactin: 0.3 – 200 ng/ml, 4.4 % and 5.3 %

- C-peptide, insulin and sex hormone-binding globulin (SHBG) were determined on either EDTA-plasma (ACTH) or serum (C-peptide, insulin and SHBG) with the Siemens Immulite® 2000 XPi immunoassay-system (Siemens Healthineers, Erlangen, Germany) by chemiluminescence with following detection ranges, inter- and intra-assay variations:
  - SHBG: 0.02 – 180 nmol/l, 5.3 % and 6.6 %
  - ACTH: 5 – 1250 pg/ml, 10 % and 9.5 %
  - Insulin : 2 – 300 µIU/ml, 5.5 % and 7.3 %
  - C-Peptide: 0.1 – 20 ng/ml, 2.3 % and 4.8 %
- Human growth hormone (hGH) and insulin-like growth factor 1 (IGF-1) were determined on serum with the IDS-iSYS immunoassay-system (Immunodiagnostic Systems GmbH, Frankfurt am Main, Germany) by chemiluminescence. According to the product inserts the detection range, inter-assay variation and intra-assay variation were as followed:
  - hGH: 0.05 – 100 ng/ml, 3.5% and 10.4 %
  - IGF-1: 10 – 1200 ng/ml, 2.9 % and 7.2 %

Laboratory parameters are accredited according to DIN EN ISO 15189:2014
